# Impact of HIV Testing and Counseling (HTC) Knowledge on HIV Prevention Practices Among Traditional Birth Attendants in Nigeria

**DOI:** 10.3390/ijerph120201969

**Published:** 2015-02-10

**Authors:** Alice Osuji, Jennifer R. Pharr, Uche Nwokoro, Anulika Ike, Christiana Ali, Ogheneaga Ejiro, John Osuyali, Michael Obiefune, Kevin Fiscella, Echezona E. Ezeanolue

**Affiliations:** 1 Prevention, Education, Treatment, Training and Research-Global Solutions-PeTR-GS, Plot 25 Liberty Estate, Independence Layout Enugu, Enugu State 400001, Nigeria; E-Mails: aaghams.petrgs@gmail.com (A.O.); unwokoro.petrgs@gmail.com (U.N.); aike.petrgs@gmail.com (A.I.); cali.petrgs@gmail.com (C.A.); mobiefune.ihv@gmail.com (M.O.); 2 School of Community Health Sciences, University of Nevada, Las Vegas, 4505 S. Maryland Parkway, Box 453067, Las Vegas, NV 89154, USA; E-Mail: jennifer.pharr@unlv.edu; 3 HealthySunrise Foundation, 8752 Castle Ridge Avenue, Las Vegas, NV 89129, USA; 4 Ministry of Health, Asaba, Delta State, 1 Onyeka Close, Asaba, Delta State 320242, Nigeria; E-Mail: drejiroaga@yahoo.com; 5 Delta State Action Committee on AIDS, No. 5 Tom Adigwu St, Off DLA Rd, Asaba, Delta State 320233, Nigeria; E-Mail: jubaisreal@yahoo.com; 6 Department of Family Medicine, University of Rochester School of Medicine and Wilmot Cancer Center, 1381 South Ave Rochester, New York, NY 14620, USA; E-Mail: kevin_fiscella@urmc.rochester.edu; 7 Department of Pediatrics, University of Nevada School of Medicine, 2040 West Charleston Boulevard, Las Vegas, NV 89102, USA

**Keywords:** HIV and AIDS, risk factors, traditional birth attendances, HIV preventive practices, HIV testing and counseling

## Abstract

Nigeria is second in the world for the number of people with HIV and has a high rate of mother-to-child transmission (MTCT). Over 60% of births in Nigeria occur outside of health care facilities, and because of this, Traditional Birth Attendants (TBAs) play a significant role in maternal and child health. It is important that TBAs be knowledgeable about HIV prevention. The purpose of this study was to determine the impact of HIV testing and counseling (HTC) knowledge on the HIV prevention practices among TBAs in Nigeria. Five hundred TBAs were surveyed. Chi-square and logistic regression were used to assess differences in HIV prevention practices between TBAs with and without HTC knowledge. TBAs with HTC knowledge are significantly more likely to engage in HIV prevention practices than TBAs without HTC. Prevention practices included: wearing gloves during delivery (*p* < 0.01), sterilization of delivery equipment (*p* < 0.01), participation in blood safety training (*p* < 0.01), and disposal of sharps (*p* < 0.01). As long as a high percent of births occur outside health care facilities in Nigeria, there will be a need for TBAs. Providing TBAs with HTC training increases HIV prevention practices and can be a key to improve maternal and child health.

## 1. Introduction

With an estimated population of 160 million people, Nigeria is second in the world in terms of the number of people living with HIV [1]. In 2013, an estimated 3.2 million people in Nigeria were HIV positive, with 400,000 of them being children [2,3]. New cases of HIV are fueled by multiple factors, including a lack of knowledge about HIV and HIV transmission, ineffective treatment of people with HIV, and inadequate access to healthcare [1]. Because of this, there has been a focus by the government and development partners to reducing the burden of HIV in Nigeria, particularly prevention of mother-to-child transmission (PMTCT). The 2010–2015 National Strategic Framework of Nigeria includes plans to increase HIV testing and expand antiretroviral (ARV) treatment to at least 80% of HIV-infected pregnant women [4].

In Nigeria, as in many developing countries, a significant percentage of births occur outside of the healthcare system. Over 60% of births take place at home and are attended by a traditional birth attendant (TBA) [5]. Some women seek out TBAs because they share the same culture or because they are trusted and respected in their community [6]. Others use the services of TBAs because of their remote location and difficulty in accessing healthcare facilities, either due to distance or cost [7]. During the birth process, there are multiple points for transmission of HIV including: mother-to-care provider, care provider-to-mother, mother-to-child, and care provider-to-community [8]. HIV can be transmitted from an HIV positive mother to a care provider if the provider does not wear personal protective equipment (PPE), such as gloves, during the birth process [9,10]. HIV can be transmitted from care provider to mother if the care provider does not use sterile equipment [9,10]. HIV can be transmitted from mother to child if the care provider does not know the HIV status of the pregnant woman and does not recommend antiretroviral therapy for HIV positive patients (mother and child) [8]. Lastly, HIV can be transmitted from care provider to the community if he/she does not properly dispose of medical waste and sharps. Because of these opportunities for HIV transmission, it is important that TBAs engage in sound HIV prevention practices.

Many health behavior theories (health belief model, social cognitive theory, stages of change theory) posit that a person must have knowledge about a disease and how to prevent the disease in order to engage in prevention behaviors [11,12,13,14,15,16,17,18,19]. Without this knowledge, a person is very unlikely to take the steps necessary to prevent the transmission of an infectious disease such as HIV [11,12,13,14,15,16,17,18,19]. This is especially true for people in the high risk occupation including relatively isolated TBAs in low-income countries. Studies have been conducted to evaluate HIV knowledge and preventive practices of TBAs in Nigeria. TBAs vary greatly in their level of training, education and knowledge of HIV prevention. Researchers have found that the majority of TBAs have either no education or primary education and/or secondary education only [5,6,20]. Few TBAs have formal, medical education and training [5,6,20]. Previous studies have shown that many lack knowledge of HIV transmission and PMTCT prior to any intervention to increase their knowledge [5,6,9,20]. Additional, TBAs have also been found to engage in HIV risk practices (*i.e.*, not wearing gloves during delivery, not sterilizing delivery equipment) [5,20]. However, researchers have found that after HIV training, TBAs significantly increased their knowledge and skills around HIV prevention [20]. Furthermore, TBAs have been identified as an important link in provision of care for pregnant women in Nigeria [9,21].

Despite previous research, little is known about the HIV prevention practices of TBAs who have received HIV testing and counseling (HTC) training. The purpose of this study was to determine the impact of HTC knowledge on the HIV prevention practices among TBAs. The research question was: Are TBAs who report having HTC knowledge more likely to engage in HIV prevention practices than TBAs who report no HTC knowledge? We hypothesized that TBAs with HTC knowledge would be more likely to engage in HIV prevention practices than TBAs without HTC knowledge.

## 2. Methods

### 2.1. Setting

The setting for this study was the 25 local government areas (LGAs) in Delta State in Nigeria. TBAs were surveyed in their various TBA’s centers, either in their maternal center or in their home if they practiced out of their home.

### 2.2. Design

The study design was cross sectional. Surveys were conducted with TBAs at a single point in time.

### 2.3. Population and Sampling

We reached out to the government to obtain a list of traditional birth attendants registered with the government and obtained a list from the Reproductive Health Department of the Ministry of Health for Delta State. Phone calls were made to each of the registered TBAs to obtain additional contacts for other TBAs in their locality both registered and unregistered. In total, 605 TBAs were asked to participate in the survey. Of the 605, forty-two declined to be surveyed, thirty were retired, and thirty-two did not complete the consent form, leaving a sample of 500 TBAs.

### 2.4. Instrument

Focus groups were held by representatives from Prevention, Education, Treatment, Training and Research-Global Solutions (PeTR-GS) of Nigeria, Delta State Primary Health, Delta State Action Committee on AIDS, University of Rochester School of Medicine, and University of Nevada School of Medicine. These focus groups were used to develop an assessment tool that allowed investigators to obtain demographic information, accessibility data, available antenatal care services, knowledge of HIV testing and counseling, clinical practices, waste disposal, blood safety measures, occupational safety and community systems. Three focus groups were held (January–February 2014), reflecting three phases of preparation for the survey. The first group included the authors (Osuji, Nwokoro, Ike, Ali, Osuyali, Obiefune, and Ezeanolue), and took place in Enugu. The second focus group was held in Delta State with the representatives from department of reproductive health, department of primary and community health (Ministry of Health) and Delta State SACA. The third focus group took place with key personnel (Community Support Specialist, Nurse Quality Improvement Specialist, Strategic Information Specialist, Program Officer and Research Coordinator) at PeTR-GS. In total, 19 people participated in the focus groups, all had post-secondary education, ten were men and nine were women and their age range was twenty to sixty years old. The survey tool was reviewed and piloted in two different LGA to determine evidence of content validity, to assess participants’ understanding and to ensure that the information obtained would be valuable in the TBA needs assessment.

The survey tool consisted of twenty-two questions that required a yes/no response. For example: “Have you ever been trained on blood safety measures?” (yes/no) and “Do you wear gloves when taking deliveries?”. Seven questions were open-ended and required a numerical answer. For example: “Total number of deliveries in the last 6 months?”. Six questions were open-ended and required a text response. For example: “How do you dispose waste?” and “How do you handle and dispose sharps?”.

### 2.5. Data Collection

A one-week planning meeting was held for our field workers to develop an action plan for conducting a TBA needs assessment. Thirteen volunteer health care workers (VHCW) residing in the community were identified and grouped into teams that included a nurse, a community support specialist and a VHCW. Teams were trained on the use of the survey instrument and assessed in competency. Each team was assigned to two LGA. Following training, each team conducted a TBA interview using the assessment tools in the local TBA language (local dialect, Nigerian Pidgin English) and recorded their responses in the assessment tool. Team members were also allowed to make some observations in the assessment tool of various responses from the TBAs. Data collection occurred over a 6-month period from March 2014 to August 2014, in twenty-five LGAs of the Delta State. Trained data entry clerks with data entry experience were used. A data specialist reviewed the data entered prior to upload. Randomly selected surveys were further reviewed for accuracy of data entry. Phone calls were completed with randomly selected surveys to review the accuracy of information.

### 2.6. Data Analysis

TBAs were asked if they had HTC knowledge. Based on their response to the question (yes or no), a dichotomous variable was created with 0 indicating that they reported HTC knowledge or 1 indicating that they did not report having HTC knowledge. Next we examined the following HIV prevention practices employed by TBAs: wearing gloves during delivery, sterilization of delivery equipment, screening patients for HIV, participation in blood safety training, disposal of sharps, and disposal of waste. For each of the HIV prevention practices, we dichotomized the variable as either yes (prevents the spread of HIV) or no. For sharps disposal and waste disposal, we consulted the WHO guidelines for minimal programs for health-care waste management. Based on these guidelines, we dichotomized the variables as either concordant with the guidelines (*i.e.*, burning or burying of medical waste; burning or encapsulating sharps) or not concordant with the guidelines (*i.e.*, dumping in the river, dumping in the bush) [22]. TBA education and training variable was dichotomized as having some formal medical training (auxiliary nurse, CHEW, nurse, midwife) or no formal medical training (junior or senior secondary school, primary school or no formal education).

SPSS 22 was used for all the analyses. We tested the hypothesis for differences in binomial proportions of HIV prevention practices of TBAs with and without HTC knowledge utilizing the following statistical tests. Chi-square tests were utilized to assess differences in demographic characteristics and HIV prevention practice proportions between TBAs with HTC knowledge and those without. The Student's t-test was used to assess differences in continuous data. Odds Ratios (ORs) were generated for the dichotomized variables of HIV preventive practices and TBA HTC knowledge using crosstabs in SPSS. Dichotomized variables were used. Adjusted Odds Ratios (AORs) were obtained through logistic regression analyses which controlled for covariates and potential confounding factors for education, certification with the state and number of deliveries in the past 6 months. Statistical analysis was accomplished using a 5% significance level.

## 3. Results

### 3.1. Characteristics of the Sample

In total, 500 TBAs were interviewed. Characteristics of the sample are provided in Table 1 and include a description of the entire sample as well as TBA with HTC knowledge and those without HTC knowledge. TBAs with HTC knowledge were more likely (*p* < 0.01) to have some formal medical training, although the percentage was low at 17.8%. They were also more likely to be registered with the government and deliver more babies (*p* < 0.01) than TBAs without HTC knowledge.

**Table 1:** Descriptive Characteristics of Traditional Birth Attendants in Nigeria.

	With HTC Knowledge *N* = 107	Without HTC Knowledge *N* = 393	Total *N* = 500	Test & *p*-Value
Category	N (%)	N (%)	N (%)	
**Education/Training**				**X^2 ^= 32.6, *p* < 0.01**
No formal training	62 (57.9)	300 (76.3)	362 (72.4)	
Primary School (6th grade)	7 (6.5)	35 (8.9)	42 (8.4)	
Junior Secondary School (Middle school)	5 (4.7)	2 (0.5)	7 (1.4)	
Senior Secondary School (High school, Diploma)	14 (13.1)	35 (8.9)	49 (10.2)	
Mid-level training or Formal Training (Auxiliary Nurse, CHEW, Nurse, Midwife)	19 (17.8)	21 (5.3)	40 (7.6)	
**Distance to nearest health facility**				**X^2 ^= 24.4, *p* < 0.01**
0–5 km	19 (17.8)	122 (31.0)	141 (28.2)	
5–10 km	40 (37.4)	159 (40.5)	200 (40.0)	
10–15 km	35 (32.7)	52 (13.2)	88 (17.6)	
>15 km	13 (12.1)	60 (15.3)	71 (14.2)	
**Estimated population of community**				**X^2 ^= 8.9, *p* = 0.06**
<5000	41 (38.3)	128 (32.6)	169 (33.8)	
5000‒10,000	53 (49.5)	173 (44.0)	226 (45.2)	
10,000‒20,000	11 (10.3)	56 (14.2)	67 (13.4)	
20,000‒50,000	1 (0.9)	16 (4.1)	17 (3.4)	
≥50,000	1 (0.9)	20 (5.1)	21 (4.2)	
**Registration with Government**				**X^2 ^= 6.7, *p* = 0.01**
Registered	56 (52.3)	151 (38.4)	207 (41.4)	
Not Registered	51 (47.7)	242 (61.6)	293 (58.6)	
**Number of Deliveries**	Mean (SD)	Mean (SD)	Mean (SD)	***T* = 3.6 *p* < 0.01**
	56.4 (64.5)	33.0 (55.2)	37.82 (57.9)	

### 3.2. HIV Prevention Practices

Prevention practices of the total sample and those with and without HTC are provided in Table 2. TBAs with HTC knowledge were more likely to have participated in blood safety training and to report screening their patients for HIV (*p* < 0.01). Additionally, a higher percentage of them wore gloves during delivery, sterilized their delivery equipment, and properly disposed of waste and sharps (*p* < 0.01) compared to TBAs without HIV knowledge. None of the TBAs with HTC knowledge disposed of waste or sharps in the river, bush or sea, while those without HIV knowledge reported disposing of waste (10.9%) and sharps (7.6%) in these locations.

**Table 2:** HIV Prevention Practices among Traditional Birth Attendants in Nigeria.

	With HTC Knowledge *N* = 107	Without HTC Knowledge *N* = 393	Total *N* = 500	Test & *p*-Value
Category	N (%)	N (%)	N (%)	
**Training in blood safety**				**X^2 ^= 8.5, *p* < 0.01**
Yes	19 (17.8)	32 (8.1)	51 (10.2)	
No	88 (82.2)	361 (91.9)	449 (89.8)	
**Screen patient for HIV**				**X^2 ^= 164.0, *p* < 0.01**
Yes	41 (38.3)	0 (0.0)	41 (8.2)	
No	66 (61.7)	393 (100.0)	459 (91.8)	
**Disposal of waste**				**X^2 ^= 45.4, *p* < 0.01**
Burn	58 (54.2)	122 (31.0)	180 (36.0)	
Bury	33 (30.8)	198 (50.4)	231 (46.2)	
Discarding in river, bush or sea	0 (0.0)	43 (10.9)	43 (8.6)	
Discard in pit or pit toilet	7 (6.5)	9 (2.3)	16 (3.2)	
Discard in flushing toilet	1 (0.9)	8 (2.0)	9 (1.8)	
Dispose at a refuse dump site	6 (5.6)	4 (1.0)	10 (2.0)	
Other (dispose, discard)	1 (0.9)	5 (1.3)	6 (1.2)	
No waste	1 (0.9)	4 (1.0)	5 (1.0)	
**Disposing of sharps**				**X^2 ^= 77.3, *p* < 0.01**
Burn	25 (23.4)	92 (23.4)	117 (23.4)	
Bury	40 (37.4)	185 (47.1)	225 (45.0)	
Dispose in a pit or pit toilet	10 (9.4)	27 (6.9)	37 (7.4)	
Discard in a river or bush	0 (0.0)	30 (7.6)	30 (6.0)	
Toilet	0 (0.0)	3 (0.8)	3 (0.6)	
Break	0 (0.0)	6 (1.5)	6 (1.2)	
Give to client	0 (0.0)	1 (0.2)	1 (0.2)	
Dispose to waste management	0 (0.0)	4 (1.0)	4 (0.8)	
Dispose at a refuse dump site	3 (2.8)	6 (1.5)	9 (1.8)	
Sharps container	22 (20.6)	4 (1.0)	23 (4.6)	
Sterilize	3 (2.8)	0 (0.0)	3 (0.6)	
Other (dispose, discard)	4 (3.7)	22 (5.6)	26 (5.2)	
No sharps	3 (2.8)	13 (3.3)	16 (3.2)	
**Gloves worn during delivery**				**X^2 ^= 33.1, *p* < 0.01**
Yes	93 (86.9)	233 (59.3)	326 (65.2)	
No	8 (7.5)	142 (36.1)	150 (30.0)	
No deliveries	6 (5.6)	18 (4.6)	24 (4.8)	
**Sterilization of delivery equipment**				**X^2 ^= 33.3, *p* < 0.01**
Yes	60 (56.1)	104 (26.4)	164 (32.8)	
No	44 (41.1)	275 (70.0)	319 (63.8)	
No equipment	3 (2.8)	14 (3.6)	17 (3.4)	

### 3.3. Odds Ratios for Prevention Practices

TBAs with HTC knowledge had significantly (*p* < 0.01) higher ORs for all of the prevention practices except disposal of waste. Compared to TBAs without HTC knowledge, they were 6.7 (CI 5.6–8.7) times more likely to screen patients for HIV, 5.4 (CI 2.7–10.7) times more likely to wear glove, 2.7(CI 1.9–3.7) times more likely to have participated in blood safety training and 2.0 (CI 1.4–2.7) times more likely to properly dispose of sharps. ORs are presented in Table 3.

**Table 3:** Odds Ratios for HIV Prevention Practices Comparing Traditional Birth Attendants HTC Knowledge to Those without HTC Knowledge.

HIV Prevention Practice	Odds Ratio (OR)	95% Confidence Interval—OR
**Screen patient for HIV**	6.7 *	**5.6–8.7**
**Glove worn during delivery**	5.4 *	**2.7–10.7**
**Sterilization of delivery equipment**	2.7 *	**1.9–3.7**
**Blood safety training**	1.9 *	**1.3–2.8**
Proper disposal of waste	1.2	0.7–2.1
**Proper disposal of sharps**	2.0 *	**1.4–2.7**

* Statistically significant at the 5% level; HTC = HIV Testing and Counseling.

### 3.4. Adjusted Odds Ratios for Prevention Practices

After controlling for covariates and potential confounding factors of education, certification with the state and number of deliveries in the past 6 months, AORs remain significant for all prevention practices except disposal of waste. Covariates of education, certification with the government and number of deliveries were also significant for the likelihood of engaging in HIV preventive practices (see Table 4). AORs greater than 1 indicate an increased likelihood of engaging in the preventive practice. The odds of wearing gloves during delivery were seven times higher and the odds of sterilizing delivery equipment were three times higher among TBAs with HTC knowledge after adjusting for education and registration with the government. The odds of completing blood safety training were 2.8 times higher among TBAs with HTC knowledge after adjusting for the number of deliveries in the past six months and being registered with the government. The odds of properly disposing of sharps were 2.2 times higher among TBAs with HTC knowledge after adjusting for education.

**Table 4:** Adjusted Odds Ratios for HIV Prevention Practices Comparing Traditional Birth Attendants with HTC Knowledge to Those without HTC Knowledge.

	B	*p*-Value	Adj. Odds Ratio	95% C.I.	
				Lower	Upper
**Wear gloves during delivery**					
**HTC knowledge**	**1.95**	*p* < 0.00 *	**7.02**	**3.23**	**15.28**
**Education**	**1.21**	*p* < 0.04 *	**3.35**	**1.09**	**10.29**
Number of deliveries (last 6 months)	0.00	*p* = 0.06	1.00	1.00	1.01
**Registered with government**	**1.35**	*p* < 0.00 *	**3.85**	**2.41**	**6.15**
**Sterilize delivery equipment**					
**HTC knowledge**	**1.14**	*p* < 0.00 *	**3.13**	**1.94**	**5.04**
**Education**	**1.14**	*p* < 0.00 *	**3.13**	**1.47**	**6.66**
Number of deliveries (last 6 months)	0.00	*p* = 0.54	1.00	1.00	1.01
Registered with government *	**0.75**	*p* < 0.00 *	**2.12**	**1.42**	**3.18**
**Blood safety training**					
**HTC knowledge**	**1.02**	*p* < 0.00 *	**2.78**	**1.42**	**5.44**
Education	−0.05	*p* = 0.92	0.95	0.32	2.79
**Number of deliveries (last 6 months)**	**0.01**	*p* = 0.03 *	**1.01**	**1.00**	**1.02**
**Registered with government**	**1.32**	*p* < 0.00 *	**3.76**	**1.97**	**7.16**
**Sharps disposal**					
**HTC knowledge**	**0.80**	*p* < 0.00 *	**2.23**	**1.38**	**3.60**
**Education**	**0.93**	*p* = 0.01 *	**2.53**	**1.26**	**5.10**
Number of deliveries (last 6 months)	0.00	*p* = 0.30	1.00	1.00	1.01
Registered with government	0.27	*p* = 0.21	1.30	0.87	1.96
**Waste disposal**					
HTC knowledge	0.13	*p* = 0.70	1.13	0.60	2.14
Education	1.25	*p* = 0.09	3.50	0.82	15.02
Number of deliveries (last 6 months)	−0.00	*p* = 0.66	1.00	0.99	1.00
Registered with government	0.11	*p* = 0.66	1.12	0.68	1.85

* Statistically significant at the 5% level; HTC = HIV Testing and Counseling.

## 4. Discussion

Findings from this study show that TBAs who have been trained in HTC and have HTC knowledge are more likely to report engaging in HIV prevention practices than TBAs who have not received HTC training and do not have HTC knowledge. This represents an important opportunity to reduce MTCT of HIV during and after birth as well as to reduce the risk of HIV infection of TBAs, mothers and the greater community. Based on health behavior theories such as the Social Cognitive Theory or the Health Belief Model, knowledge is key prerequisite to engaging in a new behavior [11,12,13,14,15,16,17,18,19]. In order for a care provider to engage in prevention practices to reduce the transmission of an infectious disease, they must have knowledge about the disease, how it is transmitted and about the behaviors needed to protect themselves and their patients from the disease. It is also important that the care provider has self-efficacy or the belief that they can successfully engage in prevention behaviors [15,16]. HTC education and training for TBAs can enhance HIV knowledge, HIV transmission routes and HIV prevention practices as well as increase TBA self-efficacy surrounding HIV prevention. This can be an effective and efficient way, in part, to combat the issue of HIV infection in SSA.

Our findings are relevant to addressing high rates of MTCT of HIV in Nigeria using TBAs. A rate limiting step in the prevention of MTCT is identifying HIV positive pregnant women [23]. In 2010, 75,000 children in Nigeria were infected with HIV and only 14% of pregnant women were tested for HIV [24,25]. When HIV positive women are identified during pregnancy and AVR therapy is provided to mother and newborn, MTCT transmission rates are reduced to less than 1% [7,26]. Approximately forty percent of TBAs with HTC knowledge reported screening their patients for HIV while none of the TBAs without HTC did so. This failure to screen presents a missed opportunity to reduce MTCT of HIV. A study by Madhivanan *et al*. found that 72% of TBAs knew that HIV could be transmitted from mother to child while only 44% knew that the risk of MTCT could be reduced [27]. However, Brennan *et al*. found that after training, TBAs were able to perform rapid saliva-based HIV testing and administer AVR therapy to HIV positive mothers and their newborns in rural Zambia [28]. Additionally, Hamela *et al*. found that, after completing a PMTCT curriculum, TBAs were able to identify HIV positive expectant mothers through screening [29]. Because the only way to identify HIV positive mothers is through screening tests, it is important that TBA be trained to use rapid HIV screening during labor and initiate AVR therapy to HIV positive mothers prior to delivery and to their newborns within 72 h of birth [30].

TBAs who are not familiar with and do not practice universal precautions increase their own risk of HIV infection and that of their patients [2,6,9]. The use of personal protective equipment (PPE), such as gloves when in contact with blood or bodily fluid greatly reduces the risk of HIV infection for care providers [6,9,10]. Sterilization of medical equipment also reduces the risk of infection of HIV negative patients [8]. Of all TBAs in this study, 65% wore gloves during delivery and 33% sterilized their delivery equipment. TBAs with HTC knowledge were significantly more likely to wear gloves and sterilize their equipment. HTC training can be an effective way to teach TBAs about universal precautions and PPE to protect themselves and their patients from infection.

This study also assessed TBA practices that may impact the greater community. Medical waste and sharps have the potential to spread HIV, as well as other infectious diseases, if not disposed of properly. Based on the WHO guidelines for minimal programs for health-care waste management, the majority of TBAs met the minimal guidelines for disposal of medical waste (burn or bury) [9]. However, TBAs with HTC knowledge were more likely to dispose of sharps in accordance with the guidelines when compared to those without HTC knowledge. Additionally, TBA with HTC where less likely to dispose of medical waste and sharps in the river, sea or bush which could improve the overall health of the population.

This is not the first study to find that the majority of TBA lack adequate knowledge about HIV and HIV prevention [22]. Of the 500 TBAs interviewed in this study, 21% reported having HTC knowledge. Findings from this study support other researchers’ findings that TBA HIV knowledge can be enhanced through training. In their study, Hamela *et al*. concluded that TBAs can be a vital component in providing PMTCT services in Malawi after completing a training curriculum in PMTCT [5,9,20,28,31,32]. Brennan *et al*. found that TBA with HIV training were able to conduct rapid HIV testing and to administer nevirapine peripartum, enhancing maternal and child care in rural communities (30). Peltzer and Henda’s pre/post evaluation of HIV training revealed that the training increased TBA HIV knowledge and decreased HIV risk practices during delivery [29].

TBAs fill an important gap in maternal services in many countries due to a number of factors including a lack of trained health providers, remote locations without access to health care facilities, cultural preferences, and poor patient-provider interactions [32]. TBAs play an important role in the birth process and maternal services and thus should be integrated into training about the prevention of HIV including MTCT.

Our study has some limitations. TBAs who were registered with the government were the initial group recruited. Contact information for TBAs not registered with the government was obtained through the initial TBAs. Many of the TBAs not registered with the government had been reluctant to complete the interview. This may have resulted in selection bias. However, over half of the TBAs were not registered with the government. HIV prevention practices were self-reported and not observed. There is the possibility of self-repot bias. The participants may have under or over reported information if they perceived the response to be socially desirable [33,34]. Despite these potential limitations*,* this study adds valuable insight into our understanding of the impact of HTC knowledge on HIV prevention practices.

## 5. Conclusions

As long as there is a shortage of skilled birth attendants and too few health care facilities in rural areas of Nigeria, there will be a need for TBAs. Providing TBAs with training and knowledge regarding HIV, HIV transmission and HIV prevention could reduce HIV prevalence in the region. Involving TBAs in PMTCT of HIV through HTC training can be a key to improving maternal and infant health and reducing the toll from HIV in Nigeria and other affected countries.

## References

[1] National Population Commission (NPC) [Nigeria] and ICF International (2014). Nigeria Demographic and Health Survey 2013.

[2] Joint United Nations Programmes on HIV/AIDS (2014). Publication of Specific Country Estimates.

[3] Joint United Nations Programmes on HIV/AIDS (2014). Children and Pregnant Women Living with HIV.

[4] National Agency for the Control of AIDS (2010). The 2010–2015 HIV/AIDS Strategic Plan.

[5] Bassey E., Elemuwa C., Anukam K. (2007). Knowledge of, and attitudes to, acquired immune deficiency syndrome (AIDS) among traditional birth attendants (TBAs) in rural communities in Cross River State, Nigeria. Int. Nurs. Rev..

[6] Balogun M., Odeyemi K. (2010). Knowledge and practice of prevention of mother-to-child transmission of HIV among traditional birth attendants in Lagos State, Nigeria. Pan. Afr. Med. J..

[7] Titaley C.R., Hunter C.L., Dibley M.J., Heywood P. (2010). Why do some women still prefer traditional birth attendants and home delivery?: A qualitative study on delivery care services in West Java Province, Indonesia. BMC Pregnancy Childbirth.

[8] UNAIDS (2007). Practical Guidelines for Intensifying HIV Prevention.

[9] Omowunmi A., Nkiru O., Yekeen R., Chinyere E., Muinat J., Segun A., Olasubomi O., Tekena H., Lateef S. (2004). Knowledge, attitudes and perceptions of HIV/AIDS among traditional birth attendants and herbal practitioners in Lagos State, Nigeria. Afr. J. AIDS Res..

[10] Board S. (2001). Risks to health care workers in developing countries. N. Engl. J. Med..

[11] Albarracín D., Durantini M.R., Earl A. (2006). Empirical and theoretical conclusions of an analysis of outcomes of HIV-prevention interventions. Curr. Dir. Psychol. Sci..

[12] Albarracín D., Gillette J.C., Earl A.N., Glasman L.R., Durantini M.R., Ho M. (2005). A test of major assumptions about behavior change: A comprehensive look at the effects of passive and active HIV-prevention interventions since the beginning of the epidemic. Psychol. Bull..

[13] Bandura A. (2000). Health promotion from the perspective of social cognitive theory. Understanding and Changing Health Behaviour: From Health Beliefs to Self-Regulation.

[14] Bandura A. (1998). Health promotion from the perspective of social cognitive theory. Psychol. Health.

[15] Bandura A. (1994). Social cognitive theory and exercise of control over HIV infection. Preventing AIDS: Theories and Methods of Behavioral Interventions.

[16] Bandura A. (1977). Self-efficacy: Toward a unifying theory of behavioral change. Psychol. Rev..

[17] Glanz K., Rimer B.K., Viswanath K. (2008). Health Behavior and Health Education: Theory, Research, and Practice.

[18] Fishbein M. (2000). The role of theory in HIV prevention. AIDS Care.

[19] Fishbein M., Yzer M.C. (2003). Using theory to design effective health behavior interventions. Commun. Theory.

[20] Perez F., Aung K.D., Ndoro T., Engelsmann B., Dabis F. (2008). Participation of traditional birth attendants in prevention of mother-to-child transmission of HIV services in two rural districts in Zimbabwe: A feasibility study. BMC Public Health.

[21] Homsy J., King R., Balaba D., Kabatesi D. (2004). Traditional health practitioners are key to scaling up comprehensive care for HIV/AIDS in sub-Saharan Africa. AIDS.

[22] Prüss A., Giroult E., Rushbrook P. (1999). Safe Management of Wastes from Health-Care Activities.

[23] Ezeanolue E.E., Obiefune M.C., Yang W., Obaro S.K., Ezeanolue C.O., Ogedegbe G.G. (2013). Comparative effectiveness of congregation- versus clinic-based approach to prevention of mother-to-child HIV transmission: Study protocol for a cluster randomized controlled trial. Implement. Sci..

[24] World Health Organization (2010). Antiretroviral Drugs for Treating Pregnant Women and Preventing HIV Infection in Infants: Recommendations for a Public Health Approach-2010 Version.

[25] World Health Organization, Unicef (2011). Global HIV/AIDS Response: Epidemic Update and Health Sector Progress Towards Universal Access: Progress Report 2011.

[26] Centers for Disease Control and Prevention (CDC) (1985). Achievements in public health. Reduction in perinatal transmission of HIV infection—United States, 1985–2005. MMWR Morb. Mortal. Wkly. Rep..

[27] European Collaborative Study (2005). Mother-to-child transmission of HIV infection in the era of highly active antiretroviral therapy. Clin. Infect. Dis..

[28] Madhivanan P., Kumar B.N., Adamson P., Krupp K. (2010). Traditional birth attendants lack basic information on HIV and safe delivery practices in rural Mysore, India. BMC Public Health.

[29] Brennan A.T., Thea D.M., Semrau K., Goggin C., Scott N., Pilingana P., Botha B., Mazimba A., Hamomba L., Seidenberg P. (2014). In‐home HIV testing and nevirapine dosing by traditional birth attendants in rural Zambia: A feasibility study. J. Midwifery Women’s Health.

[30] Hamela G., Kabondo C., Tembo T., Zimba C., Kamanga E., Mofolo I., Bulla B., Sellers C., Nakanga R.C., Lee C. (2014). Evaluating the benefits of incorporating traditional birth attendants in HIV prevention of mother to child transmission service delivery in Lilongwe, Malawi: Original research article. Afr. J. Reprod. Health.

[31] Peltzer K., Henda N. (2006). Traditional birth attendants HIV/AIDS and safe delivery in the Eastern Cape, South Africa—Evaluation of a treating programme. S. Afr. J. Obstet. Gynaecol..

[32] Peltzer K., Phaswana-Mafuya N., Treger L. (2009). Use of traditional and complementary health practices in prenatal, delivery and postnatal care in the context of HIV transmission from mother to child (PMTCT) in the Eastern Cape, South Africa. Afr. J. Tradit. Complement. Altern. Med..

[33] Gourlay A., Birdthistle I., Mburu G., Iorpenda K., Wringe A. (2013). Barriers and facilitating factors to the uptake of antiretroviral drugs for prevention of mother-to-child transmission of HIV in sub-Saharan Africa: A systematic review. J. Int. AIDS Soc..

[34] Adams A.S., Soumerai S.B., Lomas J., Ross-Degnan D. (1999). Evidence of self-report bias in assessing adherence to guidelines. Int. J. Qual. Health Care.

